# The epidemiology of firework-related injuries in the US, 2012–2022

**DOI:** 10.1186/s40621-023-00446-5

**Published:** 2023-07-04

**Authors:** Nolan M. Winicki, Ian Waldrop, Jesus V. Orozco, Daniel Novak, Nicholas W. Sheets

**Affiliations:** School of Medicine, University of California Riverside, 900 University Ave, Riverside, CA 92521 USA

**Keywords:** Epidemiology, Injury prevention, Burns, Fireworks, Recreational use, Wounds and injuries, Children, Adults

## Abstract

**Background:**

This study aimed to examine the epidemiology of firework-related injuries within a national population between 2012 and 2022, including the severity of injury by year, patient demographics, body region injured, firework type, and diagnosis category of injury.

**Methods:**

Data were collected from the Consumer Product Safety Commission’s National Electronic Injury Surveillance System, which is a representative nationwide database that collects data on consumer product-related injuries occurring in the US. Injury rates were calculated based on patient age, sex, body region injured, firework type, and diagnosis category.

**Results:**

A total of 3219 injuries, representing an estimated 122,912 firework-related injuries, were treated in emergency departments within the US from 2012 to 2022. The overall incidence rate of firework-related injuries in the study rose by over 17% from 2012 [2.61 cases per 100,000 people (95% CI 2.03–3.20)] to 2022 and [3.05 cases per 100,000 people (95% CI 2.29–3.80)]. The rate of injuries was highest in adolescents and young adults (age 20–24; 7.13 cases per 100,000 people). Men experienced firework injuries at more than double the rate of women (4.90 versus 2.25 cases per 100,000 people). The upper extremities (41.62%), head/neck (36.40%), and lower extremities (13.78%) were the most commonly injured regions. Over 20% of cases in patients older than 20 were significant injuries requiring hospitalization. Aerial devices (32.11%) and illegal fireworks (21.05%) caused the highest rates of significant injury of any firework type.

**Conclusions:**

The incidence of firework-related injuries has risen over the past decade. Injuries remain the most common among adolescents and young adults. In addition, significant injuries requiring hospitalization occur most often during aerial and illegal firework use. Further targeted sale restrictions, distribution, and manufacturing regulations for high-risk fireworks are required to reduce the incidence of significant injury.

## Background

Fireworks are commonly used worldwide to celebrate popular events, but the danger is often understated and not appreciated by the public (Ortiz Rodríguez et al. 2012; See and Lo 1994; Moore et al. 2014). Several previous studies have been performed with regard to fireworks and specific populations, injury patterns, and incidence (Ortiz Rodríguez et al. 2012; Shiuey et al. 2020; Wisse et al. 2010). Comprehensive reviews of the National Electronic Injury Surveillance System (NEISS), which produces nationwide estimates of product-related injuries in the US, have previously been limited to the policy implications of restricting the availability of fireworks or representing data from over a decade ago (See and Lo 1994; Moore et al. 2014; Berger et al. 1985). Additionally, recent studies have observed an increase in the incidence of firework-related injuries during the initial COVID-19 pandemic; however, the trends over the past decade have not been assessed (Herzog and Daley 2022; Capitelli-McMahon et al. 2022; Maassel et al. 2021).

This study aimed to examine the epidemiology of firework-related injuries within a nationally representative population between 2012 and 2022, including the severity of injury by year, patient demographics, body region injured, firework type, and category of injury.

## Methods

This study was IRB exempt and required no ethical approval because it utilized existing data that are publicly available and was recorded by the original investigator in such a manner that subjects cannot be identified, directly or through identifiers linked to the subjects.

### Data source

The data used in this study were collected from the NEISS Consumer Product Safety Commission. The NEISS is a stratified probability sample of over 100 US hospital emergency departments. The NEISS includes information extracted from medical charts, including patient demographics (i.e., age, sex, and race) and injury information including body part injured, diagnosis, geographic location where the injury occurred, product involved, and a narrative of the injury event. Race was stratified into three demographic groups (White, Black, and other) based on coding from NEISS. Firework-related injuries were identified from the NEISS using the product code 1313.

### Variables

Age was categorized in increments of 5 years, except for patients aged 60 years or older, which were combined into one group due to the small sample size. Injured body regions were categorized as head and neck, hip and lower extremities, trunk, shoulder, upper extremities, and unspecified. Diagnosis was categorized as burns, contusions/lacerations, fractures/sprains, and others.

The firework types were categorized similar to the previous studies (See and Lo 1994; Moore et al. 2014). Specifically, six groups were generated based on usage, functionality, and commonality: firecrackers, aerial devices (e.g., missiles, rockets, and aerial shells), Roman candles/fountains, sparklers/novelty devices, illegal fireworks (e.g., M80s, M100s, cherry/smoke bombs, and homemade devices), and unspecified. Firecrackers of unknown size were included in the firecracker category rather than the illegal fireworks or other/unspecified categories.

The occurrence of significant injury was defined as the patient's disposition from the emergency department being listed as treated and transferred to another hospital, treated and admitted for hospitalization or fatality. Patients who were treated/examined and then released were considered to incur non-significant injuries. Patients who left without being seen or those with an undefined disposition were not included in the injury severity analysis.

### Statistical analysis

The rates of firework-related injuries were calculated using the 2012–2022 US Census Bureau population intercensal estimates as denominators. Injury rates were calculated by age in increments of 5 years (e.g., 0–4 years, 5–9 years, and 10–14 years) with the exception of patients 60 years or older, which were combined into one category, by patient gender (male and female), body region injured (head/neck, upper trunk, upper extremities, lower extremities, and lower trunk/pubic region), firework type (aerial devices, firecracker, illegal fireworks, roman candles/fountain, sparklers/novelty devices, public, and unspecified), and diagnosis category (burns, contusions/lacerations, fracture/sprain, amputation, internal organ injury, foreign body, anoxia, and others). For all calculated estimates, a 95% confidence interval (CI) was determined, accounting for sampling error, as outlined by the CPSC.

Demographic and injury characteristics were compared between the types of fireworks using Chi-square tests. Since the NEISS is a probability sample of all hospitals with emergency departments in the US, all analyses were performed accounting for statistical weights. Statistical weights provided by the CPSC were used to calculate national injury estimates. R version 4.0 was used for all analyses, and *P*-values < 0.05 were considered statistically significant.

## Results

### Trends in injury by patient demographics and year

**Fig. 1:**
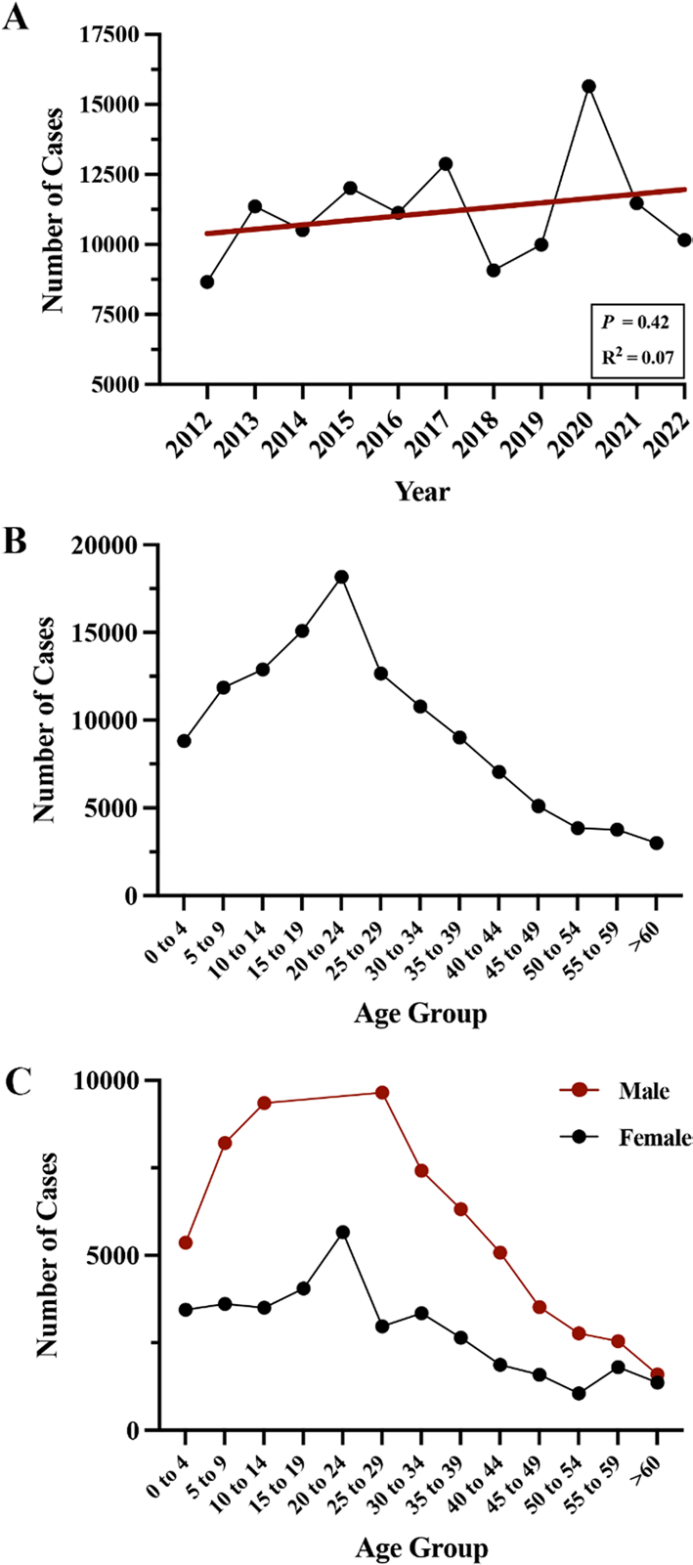
**A** Trends in the number of injuries incurred by fireworks from 2012 to 2022. **B** Incidence of firework-related injuries by age group and **C** patient sex and age group

A total of 3219 injuries, representing an estimated 122,912 firework-related injuries, were treated in the emergency departments within the US from 2012 to 2022 (Table 1). The overall incidence rate of firework-related injuries in the study rose by over 17% from 2012 to 2022 [2.61 cases per 100,000 people (95% CI 2.03–3.20) versus 3.05 cases per 100,000 people (95% CI 2.29–3.80)]. The peak incidence of firework-related injuries was in 2020 with 4.72 cases per 100,000 people (95% CI 3.68–5.77) (Fig. 1A). The relationship between the number of cases and year was not significantly linear throughout the study period (Fig. 1A; *R*^2^ = 0.07, *P* = 0.42), potentially due to a large decrease in cases in 2018–2019 followed by a spike in cases during 2020.

**Table 1:** Estimated number of firework-related injuries and incidence rates (per 100,000 persons per year) and 95% confidence intervals (CIs) by selected demographic characteristics, in the US 2012–2022

Variable	Number of cases (*N* = 3219)	National estimate (*N* = 122,912)	Rate per 100,000 (95% CI)
*Years*			
2012	256	8658	2.61 (2.03–3.20)
2013	275	11,361	3.43 (2.43–4.42)
2014	271	10,512	3.17 (2.30–4.04)
2015	296	12,011	3.62 (2.75–4.50)
2016	268	11,133	3.36 (2.50–4.22)
2017	329	12,884	3.89 (2.89–4.88)
2018	234	9081	2.74 (2.09–3.39)
2019	261	9992	3.01 (2.10–3.93)
2020	440	15,646	4.72 (3.68–5.77)
2021	338	11,471	3.46 (2.47–4.45)
2022	251	10,163	3.05 (2.29–3.80)
*Age (Years)*			
0–4	266	8805	5.40 (3.64–7.11)
5–9	420	11,857	5.44 (3.92–7.01)
10–14	434	12,887	5.88 (4.35–7.43)
15–19	402	15,086	6.73 (5.14–8.27)
20–24	427	18,182	7.13 (5.21–8.45)
25–29	307	12,661	5.25 (3.94–6.57)
30–34	238	10,776	4.79 (3.66–5.94)
35–39	201	9013	4.16 (2.99–5.33)
40–44	158	7044	3.23 (2.08–4.30)
45–49	119	5116	2.36 (1.58–3.01)
50–54	99	3861	1.35 (0.89–1.89)
55–59	71	3769	1.27 (0.81–1.72)
> 60	77	3003	1.12 (0.68–1.51)
*Gender*			
Male	2248	78,748	4.90 (3.89–5.93)
Female	971	37,522	2.25 (1.43–2.77)

The rate of injuries was highest in adolescents and young adults, with the highest incidence occurring in young adults aged 20–24 (7.13 cases per 100,000 people (95% CI 5.21–8.45) (Table 1, Fig. 1B). Men experienced firework injuries at more than double the rate of women across the study period (male: 4.90 cases per 100,000 people (95% CI 3.89–5.93) and female: 2.25 cases per 100,000 people (95% CI 1.43–2.77) (Table 1). The difference between the sexes was most pronounced in adolescence and young adulthood (Fig. 1C).

### Injuries by body region, firework type, diagnosis category, and disposition

The upper extremities (41.6%), head/neck (36.4%), and lower extremities (13.8%) were the body regions most commonly injured by fireworks (Table 2). Of the categorized firework types, injuries from sparklers/novelty devices (16.2%), firecrackers (14.9%), and aerial devices (12.2%) occurred at the highest rates (Table 2). Burns constituted the highest percentage of injuries (47.2%), followed by contusions/lacerations (21.34%), and fractures/sprains (8.5%) (Table 2). Most patients were treated or examined within the emergency room and then released (80.4%) (Table 2). However, still over 17% of patients required hospitalization and were admitted or transferred to another facility (Table 2).

**Table 2:** Firework-related injuries by body region, firework type, diagnosis category, and disposition

Variable	National estimate	Number of cases	Percentage of total (%)
*Body region*			
Head/neck	41,428	1149	36.4
Upper trunk	5558	153	4.8
Upper extremities	49,310	1314	41.6
Lower extremities	18,491	435	13.8
Lower trunk/pubic region	4391	106	3.4
*Firework type*			
Aerial devices	14,820	380	12.2
Firecracker	18,213	467	15.0
Illegal fireworks	5928	152	4.9
Roman candles/fountain	5343	137	4.4
Sparklers/novelty devices	19,695	505	16.2
Public	1443	37	1.2
Unspecified	53,925	1438	46.1
*Diagnosis category*			
Burns	57,418	1511	47.2
Contusions/lacerations	26,637	683	21.3
Fracture/sprain	10,569	271	8.5
Amputation	3216	134	4.2
Internal organ injury	3680	92	2.9
Foreign body	3861	117	3.7
Anoxia	1632	34	1.1
Other	15,468	358	11.2
*Disposition*			
Treated/examined and released	101,683	2572	80.4
Treated and transferred	8009	156	4.9
Treated and admitted/hospitalized	9457	399	12.5
Left without being seen	2302	73	2.3

### Yearly trends in all, significant, and non-significant injuries

**Fig. 2:**
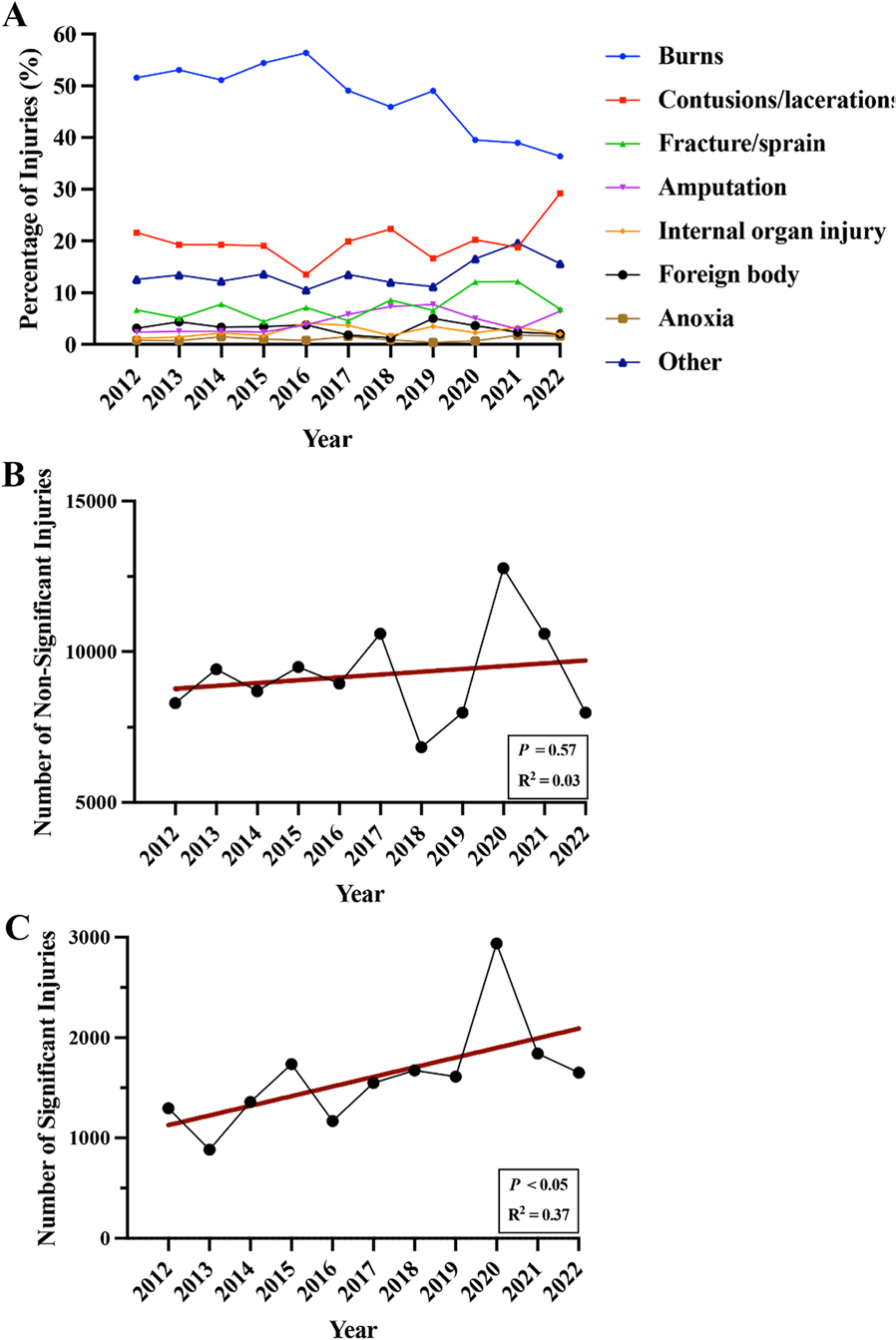
Trends in **A** all types of injuries and **B** non-significant and **C** significant injuries caused by fireworks from 2012 to 2022

The incidence of burns appeared to decrease over the study period while contusions/lacerations and fractures rose, notably in 2020 (Fig. 2A). Yearly incidence of non-significant injuries was maintained throughout the study period (Fig. 2B; *R*^2^ = 0.03, *P* = 0.57). However, the incidence of significant injuries increased from 2012 to 2022 (Fig. 2C; *R*^2^ = 0.37; *P* < 0.05). The incidence of non-significant and significant injuries peaked in 2020 (Fig. 2B and C). There was a greater than 50% increase in non-significant injuries, and over a 120% increase in significant injuries during 2020 compared to 2012.

### Significant injury by patient demographics, body region, firework type, and diagnosis category

Patients older than 20 years incurred a significant injury requiring hospitalization in over 20% of cases, while young children under 10 years of age experienced the lowest percentage of significant injury (Table 3). Men were significantly injured at almost triple the rate of women (male: 21.9% and female: 8.0%) (Table 3). Injuries to the lower trunk and pelvic region displayed the highest rate of significant injury (27.4%), followed by injuries to the upper extremities (23.1%) and upper trunk (20.9%) (Table 3). Aerial devices (32.1%) and illegal fireworks (21.1%) caused the highest rates of significant injuries for any firework type (Table 3). Most patients diagnosed with an amputation (89.2%) or fracture/sprains (40.6%) were admitted to the hospital and/or transferred to a higher level of care (Table 3).

**Table 3:** Risk of significant injury based on selected demographic characteristics, body region injured, firework type, and diagnosis category

Variable	Percentage of significant injury (%)
*Age (Years)*	
0–4	5.6
5–9	7.9
10–14	15.7
15–19	17.4
20–24	17.8
25–29	24.4
30–34	23.1
35–39	19.9
40–44	25.9
45–49	24.4
50–54	27.3
55–59	28.2
> 60	32.5
*Gender*	
Male	22.0
Female	8.0
*Body region*	
Head/neck	13.0
Upper trunk	20.9
Upper extremities	23.1
Lower extremities	10.1
Lower trunk/pubic region	27.4
*Firework type*	
Aerial devices	32.1
Firecracker	13.1
Illegal fireworks	21.1
Roman candles/fountain	13.1
Sparklers/novelty devices	5.7
Public	11.2
Unspecified	17.4
*Diagnosis category*	
Burns	10.9
Contusions/lacerations	9.1
Fracture/sprain	40.6
Amputation	89.2
Internal organ injury	26.0
Foreign body	6.1
Anoxia	20.0
Other	23.5

## Discussion

The major findings of this representative, epidemiological study are that the incidence of firework-related injuries has increased over the past decade, and significant firework-related injuries are on the rise.

Injuries from fireworks peaked during the initial stage of the COVID-19 pandemic. Previous investigations have hypothesized that this rise was due to an increase in direct consumer firework sales and a decrease in professional, public displays (Association AP 2022; Capitelli-McMahon et al. 2022; Maassel et al. 2021). Our results align with those of the previous reports and highlight that not only the incidence of injuries increased but also the number of significant injuries requiring hospitalization. Additionally, the US Consumer Product Safety Commission (CPSC) issued a report confirming that the number of firework-associated deaths increased during the pandemic lockdown (Commission USCPS 2022). While the incidence of firework-related injuries peaked in 2020, we showed that this trend has continued to increase over the past decade by over 17%. This trend directly conflicts with reports from 2000 to 2010, in which the incidence decreased by 30% over the study period (Moore et al. 2014).

Adolescents and young adults remain at the highest risk of firework-related injuries. This effect is amplified in young men, as they experienced injury rates more than double those observed in young women. Previous reports utilizing the National Emergency Department Sample have also shown a disproportionate number of head, eye, and hand firework injuries in young men, especially during June and July for Independence Day celebrations (Canner et al. Jul 2014; Bitter et al. 2021). The majority of these patients were treated at trauma and teaching centers in the midwest and south portions of the US (Canner et al. Jul 2014; Bitter et al. 2021). However, these findings do not appear to be limited to the US, as young men in Australia and China are also the most commonly injured by fireworks (Wang et al. 2014; Abdulwadud and Ozanne-Smith 1998). Firework-related injuries in the pediatric population have been well documented, and our findings support the previous conclusions (Billock et al. 2017; Witsaman et al. 2006). Specifically, reports with granular data have shown that pediatric bystanders of consumer fireworks constitute up to 13% of admitted cases, further emphasizing the importance of safer public fireworks displays rather than allowing consumer fireworks (Witsaman et al. 2006).

The upper extremities, head/neck, and lower extremities were the most commonly injured body regions by fireworks. Our findings agree with the previous literature in that the majority of cases have historically involved the hands, eyes, head, and face (Moore et al. 2014; Smith et al. 1996). The rate of significant injury requiring hospitalization was highest in injuries to the lower trunk and pelvis, followed by injuries to the upper trunk and upper extremities. While common, injuries to the head/neck were among the groups that required the lowest number of hospitalizations. Our reported incidence of significant injury to the face/neck was similar to that observed in ocular trauma investigations, which showed that one in six ocular firework traumas could cause severe vision loss (Wisse et al. 2010; Sacu et al. 2002). As burns are the most common type of injury incurred, the increased total body surface area involved during injury to the upper and lower trunk, pelvis, and extremities may contribute to the elevated hospitalization rate in these patients (Face and Dalton 2017).

Sparklers/novelty devices, firecrackers, and aerial devices caused the greatest amount of injury in our study. Additionally, aerial devices and illegal fireworks caused the highest rates of significant injury requiring hospitalization of any firework type. Compared with the previous studies utilizing the National Emergency Department Sample, our study sourced from the NEISS was able to differentiate the specific type of firework and determine the corresponding prevalence and severity of injury (Bitter et al. 2021; Gordon et al. 2023). Sparklers are the most common form of fireworks used, but display the lowest rate of injury, while the use of aerial and illegal fireworks is less frequent but incurs a higher risk of significant injury.

Firework misuse and device failure have been cited as the leading cause of firework-related injuries (Puri et al. 2009). Currently, major legislation on firework sale and personal use is decided within individual states. More restrictive laws have demonstrated a significant reduction in firework-related injuries over a 15-year period in Hawaii, while injuries increased by 100% recently after Minnesota reduced restrictions on nonexplosive and nonaerial fireworks (Roesler and Day 2007; Galanis et al. 2022). Finland and the Netherlands have been able to reduce firework-related injuries by a half through restricting hours allowed for private fireworks, the use of safety glasses, awareness campaigns, and stricter regulations on fireworks available for purchase (Faber et al. 2020) In the US, the CPSC enforces the Federal Hazardous Substances Act and the Consumer Product Safety Act to regulate the manufacture, import, distribution, and sale of consumer fireworks. However, over 30% of fireworks tested by the US CPSC were recently found to contain non-compliant components, such as fuse violations, prohibited chemicals, and overloaded pyrotechnic materials (Commission USCPS 2022). Thus, further regulatory methods on firework manufacturing and distribution are required, as well as targeted consumer education and awareness of the danger of complicated, high-risk fireworks.

The major limitation of this study is the inclusion criteria that an individual must seek emergency care. Thus, there will be patients who were harmed from fireworks but did not seek medical attention that was not included and that the reported values are underestimates of the true number of firework-related injuries. Additionally, there is no standardized injury severity score, which is commonly used in trauma departments, that is recorded in the NEISS database. Hospital disposition and mortality were used as proxies for severe outcomes in this case. Finally, the NEISS database collects only the emergency department data. Thus, the outcomes from inpatient care are not included which could impact the representation of significant injuries and mortality.

## Conclusions

In conclusion, the major findings of this representative, epidemiological study from 2012 to 2022 in the US are that the incidence of firework-related injuries has increased, and significant firework-related injuries are on the rise. Injuries remain the most common among adolescents and young adults. In addition, significant injuries requiring hospitalization occur most often during aerial and illegal firework use. Further targeted sale restrictions, distribution, and manufacturing regulations for high-risk fireworks are required to reduce the incidence of significant injury.

## Data Availability

The datasets generated and/or analyzed during the current study are available from the NEISS repository (https://www.cpsc.gov/cgibin/NEISSQuery).
